# Soil pH, total phosphorus, climate and distance are the major factors influencing microbial activity at a regional spatial scale

**DOI:** 10.1038/srep25815

**Published:** 2016-05-12

**Authors:** Haichuan Cao, Ruirui Chen, Libing Wang, Lanlan Jiang, Fen Yang, Shixue Zheng, Gejiao Wang, Xiangui Lin

**Affiliations:** 1 State Key Laboratory of Agricultural Microbiology, College of Life Science and Technology, Huazhong Agricultural University, Wuhan, 430070, P.R. China; 2 State Key Laboratory of Soil and Sustainable Agriculture, Institute of Soil Science, Chinese Academy of Sciences, Nanjing, 210008, P.R. China; 3 Novo Nordisk Foundation Center for Biosustainability, Technical University of Denmark, 2970, Hørsholm, Denmark

## Abstract

Considering the extensive functional redundancy in microbial communities and great difficulty in elucidating it based on taxonomic structure, studies on the biogeography of soil microbial activity at large spatial scale are as important as microbial community structure. Eighty-four soil samples were collected across a region from south to north China (about 1,000 km) to address the questions if microbial activity displays biogeographic patterns and what are driving forces. These samples represented different soil types, land use and climate. Redundancy analysis and nonmetric multidimensional scaling clearly revealed that soil microbial activities showed distinct differentiation at different sites over a regional spatial scale, which were strongly affected by soil pH, total P, rainfall, temperature, soil type and location. In addition, microbial community structure was greatly influenced by rainfall, location, temperature, soil pH and soil type and was correlated with microbial activity to some extent. Our results suggest that microbial activities display a clear geographic pattern that is greatly altered by geographic distance and reflected by climate, soil pH and total P over large spatial scales. There are common (distance, climate, pH and soil type) but differentiated aspects (TP, SOC and N) in the biogeography of soil microbial community structure and activity.

Microbes are perhaps the most diverse and abundant organisms on Earth, and soil microbes are the dominant engines to drive the biogeochemical cycles and a major pool of living biomass in terrestrial ecosystems^1^,^2^. At present, there is a particular interest in the link between microbial biodiversity and function in the soil. Understanding the structure–function relationships across communities over large spatial scales continues to be a major goal of ecological research^3^. Recent studies, with the aid of advanced DNA sequencing technology, have provided overwhelming evidences revealing that microbial diversity and composition display biogeographic patterns as plants and animals^4^,^5^. However, the current studies do not reach an agreement on the driving force of soil microbial diversity, *e.g.* historical processes and contemporary environmental factors. Historical events including geographic sampling locations and distance in China^6^,^7^, pH across American-continent^8^,^9^ and Great Britain^10^, and altitude in the Alps^11^, and contemporary environmental factors encompassing climate, soil moisture, soil chemistry, vegetation type, land-use type^6^,^7^,^8^,^12^ were observed as the dominant factors shaping soil microbial diversity at large spatial scales.

Although microbial structure and microbial function are intimately linked, we cannot rely entirely on our understanding of the geographical patterns in the taxonomic structure to predict the functional attributes or the functional diversity of these soil microbial communities^13^. This is because most of the soil organisms are functionally redundant and only minimum number of species is essential for ecosystem functioning^14^,^15^. It has been suggested that microbial diversity is indirectly related to biogeochemical process and estimating functional contribution from diversity requires knowledge of its status ranging from dormant to highly active^16^. Unfortunately, it is currently difficult to differentiate inactive and active cells in the soil matrix. The functional characteristics of soil microorganisms are at least as important as their biodiversity pattern in the biogeochemical studies. Up to now, only a few studies have investigated microbial function through CO_2_ respiration^16^,^17^ and soil enzyme activity^3^,^18^ at a fixed local scale, and specific nutrient inputs on global shifting of microbial communities and ecosystem functioning^19^. A recent study has challenged the traditional ecological concept by demonstrating that metabolic flexibility can be a major predictor of spatial distribution in microbial communities^20^. However, biogeographic pattern in microbial function at large spatial scales remains unknown, which is limited by the analytical methods of function.

If our first hypothesis that microbial function has biogeographic patterns is proved true, we would like to know the driving force of this pattern by historic events or contemporary environmental factors, or both. It is widely assumed that the soil microbial function is determined primarily by the environmental factors, however, some studies showed that biochemical function was related to community composition of microbes in soil at a local scale, and both microbial community structure and their function were influenced by soil pH, temperature, moisture and nutrient availability^3^,^16^,^21^,^22^. At large spatial scales, the structure-function relationships are confounded. For example, both structure and enzyme activities of soil microbial communities differed due to the environmental factors such as forest types and the regional climates^18^. By contrast, soil fungi were endemic to bioregions, whereas soil extracellular enzyme activity showed stochastic pattern^3^. This suggests that microbial function can be as similar as geographic pattern of diversity and composition, and dissimilar influences of historical events versus environmental factors are related to the sampling effort or spatial scale^23^.

Soil microbial functioning can be represented by soil microbial activity, as the comprehensive activity of microbial communities manifests soil functioning. For decades, many approaches have been applied to study soil microbial activity such as soil basal respiration^21^,^22^,^24^, substrate-induced respiration^25^,^26^,^27^ and soil enzymes^3^,^18^,^28^. The approaches based on microbial growth such as respiration and substrate utilization are the most advantageous and allow simultaneous quantitative estimation of microbes in soil at a global scale^29^. Soil microorganisms can be classified as active (0.1–2%), potentially active (40–60%), dormant and dead cells. The active microorganisms are involved in the ongoing utilization of easily available substrates^29^. The potentially active microorganisms, so called “resting cells”, are expending energy to maintain a state of metabolic alertness and can switch to utilization of substrates within minutes to a few hours^30^. As substrate-induced-activity can represent the potential activity of roughly half of the microbes in targeted soils, we employed substrate-induced-calorimetry in the present study to represent microbial activity. Calorimetry measures heat-dissipating process of cell growth and interactions between molecules. Soil microbial activity during a long period of time can be sensitively monitored without disruption of soil system by this method, providing qualitative and quantitative indicators of microbial growth in live^31^. Calorimetric parameters are well correlated with soil microbial respiration rate, biomass, and activities of many soil enzymes^25^,^26^,^27^,^32^,^33^,^34^. Calorimetry has been applied in the studies of the influence of nutritional status, fertilizers, landscape and pollutants on microbial activity and is a powerful tool for assessing soil microbial activity and soil quality^31^,^35^,^36^.

In the present study, we collected 84 soil samples across a regional scale (about 1,000 km) in China, covering different soil type, climate, vegetation, and chemical properties. We build such a system to examine (1) our hypothesis that soil microbial activity displays biogeographic patterns at large spatial scales, (2) the controlling factor of microbial activity, *e.g.* historical events and environmental factors, and (3) how common microbial community structure and function are linked.

## Results

### Soil chemical properties

**Figure 1:**
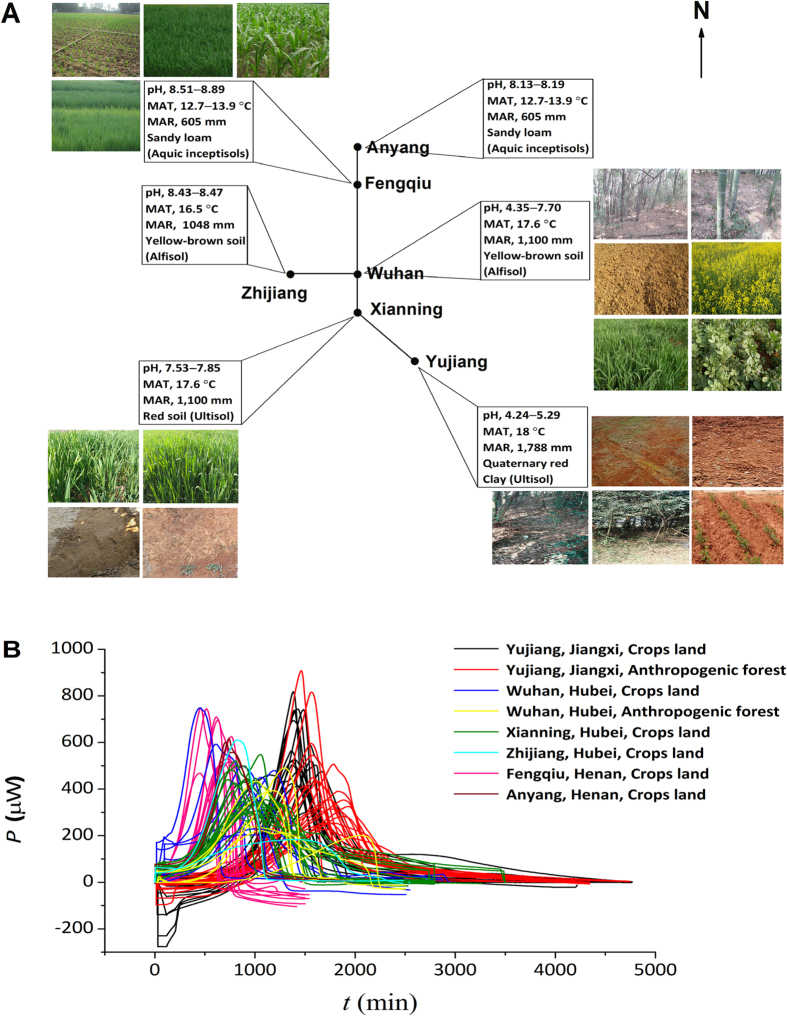
Sampling sites (**A**) and calorimetrical characterization of 84 soil samples amended with glucose and ammonium sulphate (**B**). In these curves thermal power (μW) is plotted against time (min). MAT, mean annual temperature; MAR, mean annual rainfall.

Due to all of soil samples were collected from traditional crops-landed area and anthropogenic forest, the soil pH did not show extreme acidic or alkaline value ranging from 4.2 to 8.9 (Fig. 1A and Table 1). From south to north area, mean annual temperature (MAT) and mean annual rainfall (MAR) gradually decreased, whereas soil pH increased except the soil pH in Wuhan at central China varying greatly (pH 4.35–7.7). Soil types included red soil in south China, yellow-brown soil in central China and sandy loam soil in north China.

Compared to soil organic C (SOC, 5.2–32.3 g kg^−1^), total N (TN, 0.5–1.9 g kg^−1^) and total P (TP, 0.3–1.3 g kg^−1^), mineral N (MN, 2.0–145.0 mg kg^−1^) and available P (AP, 2.0–215 mg kg^−1^) shifted greatly in different soils (Table S1). In particular, the higher contents of SOC and TN occurred in Xianning and Wuhan in Hubei province, and higher content of TP in Fengqiu and Anyang in Henan province but lower in Yujiang (Table S1). Notably, a few samples (no. 22–24, 31–33 and 70) had extremely low contents of available P (less 2.5 mg kg^−1^). The ratio of SOC/TN was more stable than the ratio of SOC/TP, SOC/AP, TN/TP and TN/AP for all soil samples.

### Substrate-induced soil microbial activities and correlations with soil profile

Recorded power-time curves were obtained from incubated soils amended glucose and ammonia, presenting a growing process of microorganisms and substrate-induced soil microbial activity (Fig. 1B). The peak power (*P*_max_), peak time (*t*_max_), rate of heat output (*Q*_T_/*t*), total heat output (*Q*_T_) and constant of growth rate (*k*) were calculated from the power-time curves and used to the following correlation analysis. In general, lower *t*_max_, shorter lag phase, higher *Q*_T_/*t* and *P*_max_, and greater *k* indicated the high microbial activity in a microcalorimetric analysis^32^,^34^,^35^. The *t*_max_ of soils increased with higher MAT and MAR, and decreased by soil pH from north to south area (Fig. 1). The highest *t*_max_ value indicating the lowest microbial activity was observed in the acidic soils from Yujiang in contrast to the lowest *t*_max_ in alkaline soils from Fengqiu, while the median value of *t*_max_ appeared in near-neutral soils from Xianning under middle level of MAT and MAR. However, the *t*_max_ values in soils from Wuhan with pH 4.4–7.7 varied greatly even under the same climate and soil type. The *P*_max_ changed greatly in the soils from the same site (Fig. 1 and Table S1).

**Figure 2:**
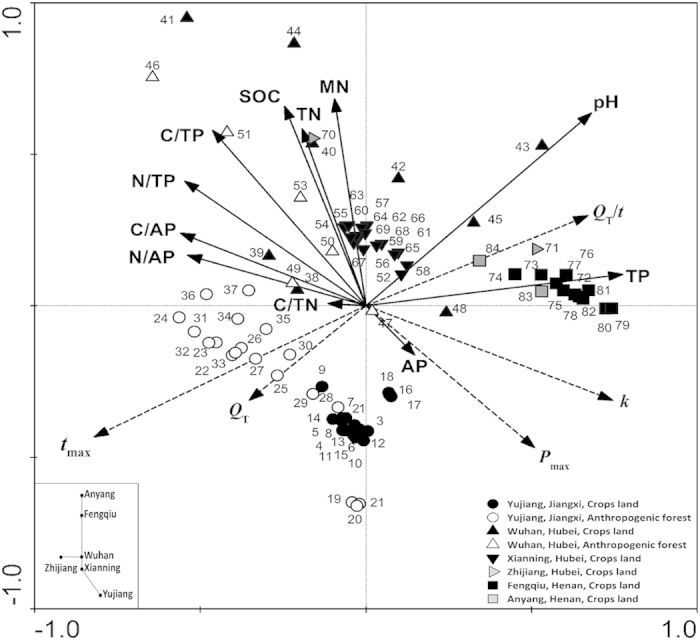
Redundancy analysis of the relationships among calorimetric parameters, soil chemical properties and sampling sites. Chemical properties SOC, soil organic C; TN, total N; MN, mineral N; TP, total P; AP, available P. *Q*_T_ is total heat output. The *P*_max_ and *t*_max_ are the power and time to reach the maximum of the peak, respectively. *k* is the growth rate constant. *Q*_T_/*t* is rate of heat output.

Redundancy analysis (RDA) displayed relationships of calorimetric parameters and environmental and geographic factors (Fig. 2). The *Q*_T_/*t* had the opposite pattern to *t*_max_, indicating lower activity in soils from Yujiang and higher activity in soils from Xianning and Fengqiu. The *t*_max_ negatively but *Q*_T_/*t* positively increased by the soil pH and TP, respectively. Both *k* and *P*_max_ were correlated with SOC, MN, AP, TN and ratios of C/P and N/P. In particular, RDA clearly indicated that soil samples from various locations were independently distributed from one to another. These results demonstrate that microbial activities are intimately linked with soil chemical properties, locations and soil types at a regional spatial scale.

### Geographic differentiation of soil microbial activities

**Figure 3:**
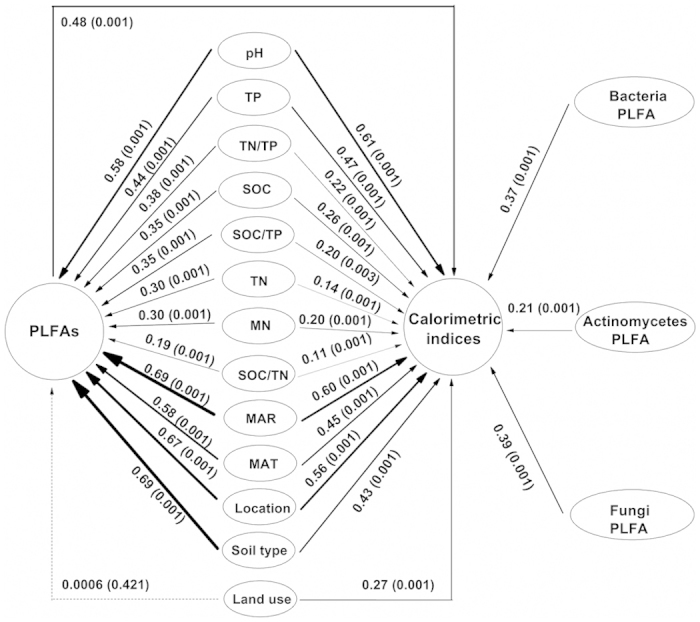
Mantel path analysis linking correlations between environmental factors, calorimetric indices and PLFAs. Solid lines are partial Mantel correlation coefficients, while dashed lines are pure-partial Mantel correlation coefficients, conditional on all other variables. Where Mantel correlations are significant, line width is proportional to the correlation coefficient, and P values are in parentheses. PLFA, phospholipid fatty acid. Chemical properties SOC, soil organic C; TN, total N; MN, mineral N; TP, total P; AP, available P. MAT, mean annual temperature; MAR, mean annual rainfall.

**Figure 4:**
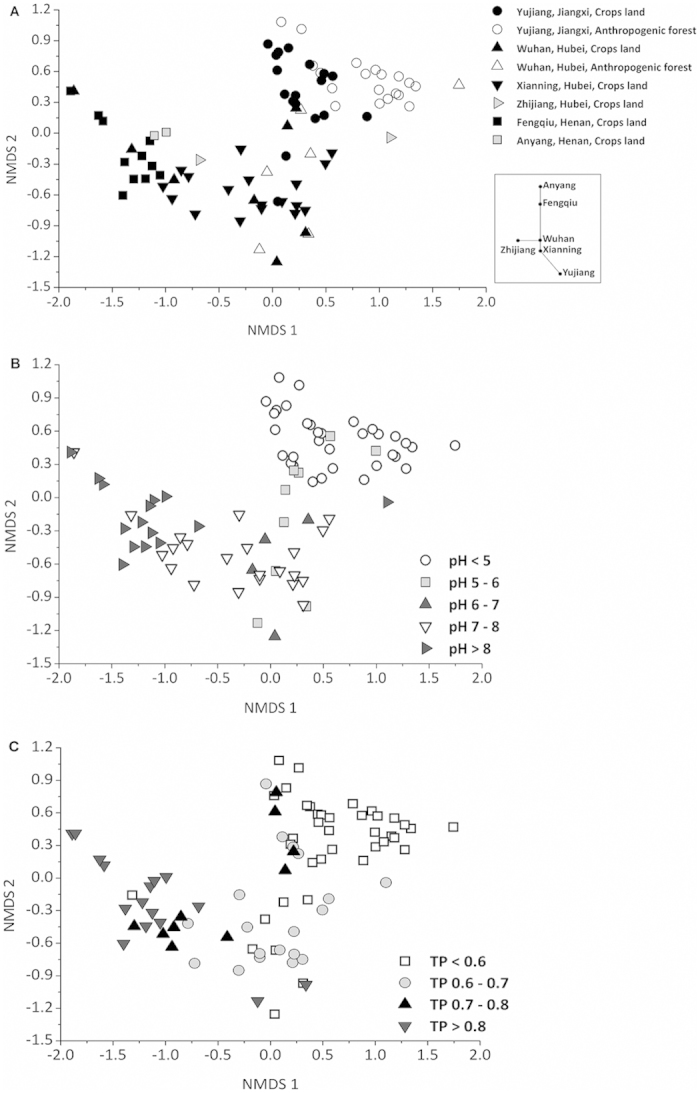
Nonmetric Multidimensional Scaling (NMDS) of calorimetric parameters (*t*_max_, *P*_max_, *Q*_T_, *k* and *Q*_T_/*t*) from 84 soil samples. Plot (**A**) is overlayed with site and land use category, (**B**,**C**) with pH and TP category, respectively. The R^2^ values between ordination distance and distance in the original space are 0.75 and 0.24 for axis 1 and axis 2, respectively.

Mantel test confirmed that chemical properties especially pH (r = 0.61) and TP (r = 0.47) were correlated with total microbial activities in soils (Fig. 3). Besides soil chemical properties, other environmental and geographic factors including MAR (r = 0.60), MAT (r = 0.45), location (r = 0.56), soil type (r = 0.43) and land use (r = 0.27) effected total microbial activities (*P* < 0.001) (Fig. 3). According to nonmetric multidimensional scaling (NMDS) plots, we further confirmed that soil pH (54% of NMDS1), TP (48%), MAT (61%) and MAR (54%) were the strongest predictors of microbial activities (Table S2). Furthermore, we observed a general clustering of soil microbial activities within soil sites and the longer distance corresponding to the greater differentiation of activities (Fig. 4A). The differentiation in microbial activities between pairwise sites was larger than that between land uses (crops and forest) within the same site in Yujiang or Wuhan. In addition, soil microbial activities were clearly clustered in response to pH gradient (Fig. 4B) and TP (Fig. 4C), particularly from soils with higher contents (>0.8 g kg^−1^) and lower contents (<0.6 g kg^−1^) of TP. Taken together, soil microbial activities were clearly separated from areas of Jiangxi, Hubei and Henan provinces despite occasional overlap (Figs 2, 3, 4). These differences can be explained by distance, climate, soil pH, soil TP, soil type and land use.

### Microbial community structure correlated with soil profile and its biogeographic traits

**Figure 5:**
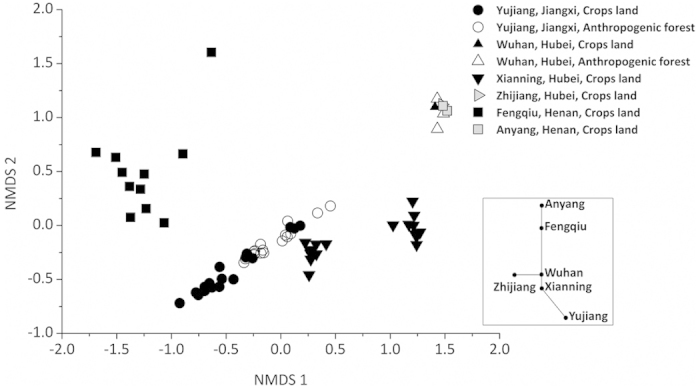
Nonmetric Multidimensional Scaling (NMDS) of PLFAs in 72 soil samples. The R^2^ values between ordination distance and distance in the original space are 0.86 and 0.14 for axis 1 and axis 2, respectively.

In order to understand the geographic patterns of microbial community structure, we also investigated the link between microbial structure, represented by phospholipid fatty acids (PLFAs), and environmental and geographic factors. The largest amount of PLFAs for bacteria and fungi was observed in soil samples from Fengqiu, followed by Xianning, while the lowest amount of PLFAs appeared in Yujiang, especially for crop land (Table S1). In contrast, PLFA amount of actinomycetes was the highest in soil samples from Xianning, followed by Fengqiu and the lowest from Yujiang. Soil pH, SOC, TN and TP were all significantly correlated with bacteria, actinomycetes, fungi and total microorganisms (Table 2). However, the impact of specific factor on different microbial biomass varied greatly. For example, soil pH is the factor to strongly influence bacteria and fungi in contrast to TN and MN mostly influencing actinomycetes. Mantel test also confirmed that chemical properties, in particular pH (r = 0.58, *P* < 0.001), were correlated with total microbial PLFAs in soils (Fig. 3). Other chemical properties including TP, SOC, TN and MN had less correlation with total PLFAs than pH (Fig. 3). NMDS further confirmed that soil chemical properties affected the differentiation of PLFAs, which were explained by soil pH (58%), followed by MN (54%), SOC (42%), SOC/TP (38%) and TN (33%) along the NMDS1 (0.86) (Fig. 5 and Table S2).

NMDS showed that PLFA composition had characteristics of regional affinity (Fig. 5). The clustering of PLFA composition from soils was separated between pairwise sites, while two sub-clusters appeared in Yujiang (land use) and Xianning (season), respectively. Moreover, PLFA composition had a good correlation with geographic factors such as MAR (r = 0.69), soil type (r = 0.69), location (r = 0.67) and MAT (r = 0.58) (*P* < 0.001) (Fig. 3).

### The link between microbial activity and community

In order to gain a deeper insight into the link between PLFAs and microbial activities, Mantel analysis was conducted, showing total PLFAs (r = 0.48, *P* < 0.001) and different microbial groups were correlated with calorimetric parameters from 72 soil samples (Fig. 3). Bacteria (r = 0.37) and fungi (r = 0.39) were better than actinomycetes (r = 0.21) correlated with total microbial activity (*P* < 0.001). Moreover, the relationship between microbial groups and specific thermal parameter was analyzed (Table 2). Total PLFAs had a greater correlation with the most predominant parameters of microbial activity such as *t*_max_ (R^2^ = 0.66, *P* < 0.001) and *Q*_T_/*t* (R^2^ = 0.62, *P* < 0.001) than other calorimetric parameters. In contrast, PLFAs of bacteria and fungi had the same correlating pattern as total PLFAs with *t*_max_ and *Q*_T_/*t* (Table 2 and Fig. 3).

## Discussion

### Biogeographic patterns in microbial activity

Microbial structure is intimately linked with function, but the relation between structure and function has different temporal and spatial traits because only minimum number of microbes are active and functioning^2^,^14^,^15^. Microbial activity is intrinsic to Earth system reflecting ecosystem dynamics and energy exchange between the biosphere and the atmosphere^3^,^20^. As a result, understanding the microbial activity is at least as important as understanding the microbial community structure. This study is the first quantitative examination of the impact of the environmental and geographic factors on microbial activity at a large spatial scale. We have found that microbial activity is delineated strongly by geographic regions within a regional scale (about 1,000 km) (Fig. 4). The distribution of microbial activity is related to the horizontal spatial gradient such as climate gradient (MAR and MAT) and soil type represented by the different sampling locations (Figs 3 and 4), which is mainly explained by soil chemical properties such as pH and TP (Figs 2 and 4, Table S2). To further validate the effect of geo-distance on microbial activity, a few soil samples from Anyang (distance-close to Fengqiu) and Zhijiang (close to Wuhan) were used to this case. Interestingly, they all clustered into geo-distance-near group of Fengqiu and Wuhan, respectively, except for one sample from Zhijiang (Figs 2 and 4) because of extremely low content of available P (Table S1)^36^. By contrast, land uses had a less correlation with microbial activity than climate gradient, location and soil type. For example, microbial activities of two land uses (crops land and anthropogenic forest) within Yujiang showed a lower differentiation than that between pairwise sampling sites (Figs 2, 3, 4). Similarly, soil properties better explained the differences in microbial communities than land use effects across a range of European field sites^37^.

In order to observe the influence of the season on microbial activity, 18 soil samples from Xianning were collected in spring and autumn, respectively. Interestingly, all 18 soil samples were clustered together, clearly separating from the soil samples from other provinces of Jiangxi and Henan (Figs 2 and 4). It only showed an overlap with soil samples from distance-close site of Wuhan. This indicates that season has less influence on geographic distribution of substrate-induced microbial activity at a large spatial scale. We also investigated the soil microbial activities with pH gradient at a small spatial scale. Fourteen soil samples were collected from Wuhan with various pH from 4.35 to 7.7 (Table S1). The microbial activities of all soil samples from Wuhan were grouped together, clearly separated from soil samples from other provinces of Jiangxi and Henan (Figs 2 and 4), indicating soil pH greatly influences differentiation of microbial activity in a close distance but is less than that of geo-distance at a large spatial scale.

Furthermore, we used an approach of substrate-induced microbial activity to study the biogeographic pattern of microbial activity in detail, which is different from the approach depending on taxonomic structure to predict the functional attributes and this method can represent the potential activity of roughly half of the microbes in targeted soils^29^. Recent studies provide overwhelming evidence that both historical events such as geographic sampling locations and contemporary environmental factors including soil pH, climate, moisture, soil chemistry, vegetation type, land-use type and altitude shape soil microbial diversity and composition at large spatial scales^4^,^5^,^6^,^7^,^8^,^9^,^10^,^11^,^12^. Similarly, the distribution of microbial activity is mainly linked with both historic events such as location, geo-distance and current environmental factors encompassing climate gradient (MAR and MAT), soil type, soil chemical properties of pH and TP (Figs 2 and 4, Table S2). This supports the ecological concept that metabolic flexibility is a major predictor shaping spatial distribution in microbial communities^20^. Therefore, the strong effects of distinct geographic location, climate and soil profiles on microbial activity indicate a strong biogeographic provincialism of microbial activity in soils at a large spatial scale.

### Inconsistent relationship between microbial community and activity at large spatial scales

Mantel analysis showed total PLFAs were correlated with total calorimetric results (r = 0.48, *P* < 0.001) (Fig. 3). On specific calorimetric parameters, PLFAs had good correlations with *t*_max_ (R^2^ = 0.66), *Q*_T_/*t* (R^2^ = 0.62) and *k* (R^2^ = 0.34) (*P* < 0.0001), and the biomass of different microbial groups such as bacteria and fungi had the same trend (Table 2). In addition, both microbial activity and microbial community are influenced by climate (MAR and MAT), location, soil type and soil chemical properties but at different degree (Fig. 3 and Table S2). These results indicate that the microbial structure and activity of ecological communities are linked together to certain extent^2^. However, this correlation is not consistent, suggesting the existence of functional redundancy of microbial communities^3^,^16^.

Notably, microbial activity and community structure were both strongly shaped by soil pH (Table S2, Figs 4 and 5). Soil pH is affected by the mineralogy, climate, weathering, and agro-management, and thus pH is an integrated indicator of soil and associated with geography^6^,^38^. Soil pH was the main driver determining bacterial diversity, richness and community composition at large spatial scales^8^,^9^,^10^, even across the 180-m distance of artificial trial of pH gradient^8^. Soil pH also had significant effects on soil enzyme activities^39^, respiration and soil metabolic quotient (*q*CO_2_) at specific sites^21^,^22^. Our results clearly manifest that both soil microbial composition and microbial activity are strongly affected by soil pH which is a common aspect of biogeography.

The other chemical properties had less impact on microbial activity than pH, and they showed different degrees of influence on microbial activity and microbial community at large spatial scales. In grasslands across the globe, elevated N and P inputs shifted the abundances of soil microbial communities and representative functional genes^19^, suggesting the large impacts of soil chemical properties on microbial community and functioning. Soil TP had larger influence on microbial activity than community structure, while soil SOC, MN and TN more affected community structure than microbial activity (Table S2, Figs 4 and 5). This is probably due to the stability of pH (slightly disturbed by human) and the fluctuation of nutrients (strongly disturbed by human) in a specific area of agricultural soils. In agricultural soil, SOC and N are more disturbed by human activity and partially erase the effects of geographic and evolutionary events, and thus have impacts on soil microbial community^6^ and activity in a local spatial scale. Alternatively, microbial activity is measured by C-N-induced calorimetric method, resulting in the mask of true picture of SOC and N affecting microbial activity.

In general, the limitation of P negatively affected the growth of crops and microbial activity in terrestrial ecosystems^40^,^41^. Soil microorganisms did not grow when available P was less than 0.7 mg kg^−1^ in soil whereas microbial growth was stimulated by the addition of available P^36^. In this study, when the available P reached 2.0 mg kg^−1^, soil microorganisms grew, but the heat output curve showed lower *P*_max_ and longer *t*_max_ (sample No. 70) (Fig. 1B), resulting in the deviation of this soil sample from their cluster (Figs 2 and 4). At large spatial scales, inorganic P availability was strongly associated with microbial metabolic quotients (*q*CO_2_) in global soils^17^. By contrast, P limitation in soil may not influence the detection of PLFAs, even when microbes are in a dormant state. However, these dormant cells affect the determination of activity^29^. As such, total P could greater affect microbial activity than community structure at a large spatial scale.

Spatial scale is an essential predictor to drive the biogeographic pattern of community structure^4^,^23^. At intermediate scales (10–3000 km), both historical events and current environmental conditions influence composition^23^. In this case, there is a strong effect of location, climate (MAR and MAT), soil type and soil chemical properties on microbial activity and community structure at a regional spatial scale (about 1000 km) despite a certainly different degree of effects as mentioned above (Figs 3, 4, 5). Therefore, the microbial activity and microbial community structure are linked together by sharing a common (distance, climate, soil pH and soil type) but differentiated aspect (such as soil TP, SOC and N) of geographic provincialism.

In conclusion, like the soil microbial diversity and composition, the strong effects of distinct location and soil profile on substrate-induced microbial activity display a clear biogeographic pattern of microbial activity in soils across a regional spatial scale. To our knowledge, no such investigation has been performed previously. The distribution of microbial activity is mainly linked with both historic events such as location and geo-distance and current environmental factors encompassing climate gradient (MAR and MAT), soil type, soil chemical properties of pH and TP. Other soil nutrients such as SOC and N that can be greatly disturbed by human activity have less impact on microbial activity. Location, climate (MAR and MAT), soil type and soil pH also drive the biogeographic pattern of microbial community structure. Moreover, PLFA is correlated with calorimetric parameters. This investigation indicates that there is a common aspect of biogeography such as distance, climate, soil pH and soil type to soil microbial community and activity over large spatial scales, although certain environmental factors such as TP, SOC and N have different impacts on them. The present results clearly point the way to elucidate global biogeography of soil microbial activity for future studies.

## Methods

### Site description and soil sampling

Soil samples were collected from different areas including arable land and anthropogenic forest in three provinces (Jiangxi, Hubei and Henan) across south to north China (Table 1 and Fig. 1A). Briefly, the distance of sampled sites as the crow flies is about 1,000 km with a wide range of soil pH (4.2–8.9) from south to north areas. Yujiang, located in Jiangxi Province, south China, has a subtropical monsoon climate, with a MAT of 18 °C and a MAR of 1,788 mm. Wuhan, Xianning and Zhijiang, Hubei province, central China, have a typical subtropical monsoon climate. The MAT is 17.6 °C and the MAR is 1,100 mm. Fengqiu and Anyang, located in Henan province in north China, are continental temperate monsoon climate with MAT of 12.7–13.9 °C, and MAR of 605 mm. The soils from Jiangxi province are acid Quaternary Red Soil, which are widely distributed in southeast China. The soils from Fengqiu and Anyang are typical sandy loam (aquic inceptisols), which account for about 20% of all Chinese soils. In contrast, the soils in Hubei province are more diverse than other two provinces with red soil from Xianning and yellow-brown soils from Wuhan and Zhijiang (Table 1).

All soil samples were collected from upland in plain area avoiding elevation gradient. At each plot, soil was collected at a depth of 1–15 cm from 5–10 locations within a given plot of 100 m^2^ and composited into a single bulk sample. They were air-dried, homogenized by sieving to less than 2 mm to separate roots and large object, and stored in polyethylene bags at 4 °C.

Soil chemical properties of pH, soil organic C (SOC), total N (TN), total P (TP), mineral N (MN) and available P (AP) were measured in each soil sample by using methods as described^36^.

### Microcalorimetric measurement

Metabolic activities of soil microorganisms were evaluated with a third generation thermal activity monitor (TAMIII, Järfälla, Sweden)^42^. All soil samples were first placed at 28 °C for 6 h and then submitted to microcalorimetric measurement^34^. A solution containing 5.0 mg of glucose and 5.0 mg of ammonium sulphate was added to 1.0 g soil sample in a 4-ml sterilized steel ampoule. To avoid the negative impact of soil water on microbial activity, the level of moisture was kept constant for all soil samples during growing process of soil microorganisms^43^. The temperature of the calorimeter system and the isothermal box were controlled at 28 °C. The power-time curve of microbial growth was continuously monitored and recorded on a computer. The thermodynamic parameters, namely, constant of growth rate (*k*), peak power (*P*_max_), peak time (*t*_max_), and total heat output (*Q*_T_) were obtained by integrating the power-time curves^35^,^44^. In detail, *Q*_T_ is the total heat output during organic material consumption and it reflects the activities of soil microorganism^25^, the rate of heat output (*Q*_T_/*t*) is ratio of *Q*_T_ to the total time of metabolic process. The *P*_max_ and *t*_max_ are the power and time to reach the maximum of the peak, respectively^35^,^44^. The *k* is the constant of growth rate and was calculated from the slope of semi-logarithm of the exponential phase^43^.

### Phospholipid fatty acid (PLFA) analysis

PLFAs were detected by Gas Chromatography-Mass Spectrometer (GC-MS)^45^,^46^,^47^. All soils except some samples from Wuhan, totally 72 samples, were included in this analysis. The total quantity of individual fatty acid methyl esters was determined using methyl nonadecanoate (19:0) as an internal standard^47^.

The fatty acids 15:0, 16:0, cy17:0, 17:0, 18:0, cy19:0, 16:1*ω*7, and 16:1*ω*5 were used as bacterial identification markers^48^,^49^. The methylated, branched, saturated fatty acids of 10Me18:0 and 10Me19:0 represented actinomycetes^50^. The fatty acids of 18:2*ω*6 and 9c were used as indicators for fungi^46^,^51^.

### Statistical analysis

All results were expressed on a soil oven-dry weight basis (105 °C, 24 h). Statistical analyses were carried out with SPSS 17.0. Redundancy analysis (RDA), a multivariate direct gradient analysis method, was calculated by Canoco version 4.5 to elucidate the relationships among microcalorimetric parameters, chemical properties, and sampling sites. Correlations among soil chemical properties, microcalorimetric parameters and phospholipid fatty acid (PLFA) composition were examined by using linear regressions with a Pearson correction for multiple comparisons. Mantel analysis was performed among environmental factors, microbial activity and PLFA composition^8^. Nonmetric multidimensional scaling (NMDS) plots of microbial activities and community structure were conducted in PC-ORD 5.0 by using the Sorensen distance metric, with Monte Carlo test (999 randomized runs) to determine significance. Regression analysis was then performed to explain the variability along with NMDS axes affected by environmental factors including soil chemical properties and climate.

## Additional Information

**How to cite this article**: Cao, H. *et al.* Soil pH, total phosphorus, climate and distance are the major factors influencing microbial activity at a regional spatial scale. *Sci. Rep.* **6**, 25815; doi: 10.1038/srep25815 (2016).

## Supplementary Material

Supplementary Information

## Figures and Tables

**Table 1:** Biogeography properties.

Soil samples	Sampling sites	Latitude & Longitude	Land use	Soil type	Climate
1–18	Yujiang, Jiangxi province	28°13′N, 116°54′E	Crops land	Quaternary red Clay (Ultisol)	Subtropical monsoon
19–37	Yujiang, Jiangxi province	28°13′N, 116°54′E	Anthropogenic forest	Quaternary red Clay (Ultisol)	Subtropical monsoon
38–45	Wuhan, Hubei province	30°29′N, 114°22′E	Crops land	Yellow-brown soil (Alfisol)	Subtropical monsoon
46–51	Wuhan, Hubei province	30°29′N, 114°22′E	Anthropogenic forest	Yellow-brown soil (Alfisol)	Subtropical monsoon
52–69	Xianning, Hubei province	29°5′ N, 114°15′E	Crops land	Red soil (Ultisol)	Subtropical monsoon
70–71	Zhijiang, Hubei province	30°26′N, 110°30′E	Crops land	Yellow-brown soil (Alfisol)	Subtropical monsoon
72–82	Fengqiu, Henan province	35°00′N, 114°24′E	Crops land	Sandy loam (Aquic inceptisols)	Continental temperate monsoon
83–84	Anyang, Henan province	35°32′N, 114°50′E	Crops land	Sandy loam (Aquic inceptisols)	Continental temperate monsoon

**Table 2:** The soil PLFAs correlated with soil chemical properties and calorimetric parameters.

		Bacterial PLFAs		Actinomycetes PLFAs		Fungal PLFAs		Total PLFAs	
		R^2^	*P*	R^2^	*P*	R^2^	*P*	R^2^	*P*
Soil chemical properties	pH	0.78	<0.001	0.54	<0.001	0.42	<0.001	0.80	<0.001
	SOC	0.21	<0.001	0.60	<0.001	0.07	=0.032	0.16	=0.001
	TN	0.20	<0.001	0.66	<0.001	0.07	=0.027	0.12	=0.005
	MN	0.43	<0.001	0.76	<0.001	–	>0.5	0.41	<0.001
	TP	0.36	<0.001	0.12	=0.004	0.34	<0.001	0.34	<0.001
	AP	–	>0.05	–	>0.05	–	>0.5	–	>0.50
	SOC/TN	–	>0.05	–	>0.05	–	>0.5	–	>0.50
	SOC/TP	–	>0.05	0.17	<0.001	0.22	<0.001	0.09	=0.014
	SOC/AP	–	>0.05	–	>0.05	0.05	=0.035	–	>0.5
	TN/TP	0.07	=0.033	0.23	<0.001	0.26	<0.001	–	>0.5
	TN/AP	–	>0.05	–	>0.05	0.05	=0.039	–	>0.5
Calorimetric parameters	*P*_max_	0.13	=0.003	0.10	=0.004	–	>0.5	–	>0.5
	*t*_max_	0.60	<0.001	0.35	<0.001	0.42	<0.001	0.66	<0.001
	*Q*_T_	0.13	=0.001	–	>0.5	0.27	<0.001	0.18	<0.001
	*k*	0.20	<0.001	–	>0.5	0.65	<0.001	0.34	<0.001
	*Q*_T_*/t*	0.52	<0.001	0.22	<0.001	0.63	<0.001	0.62	<0.001

PLFA, phospholipid fatty acid. Chemical properties SOC, soil organic C; TN, total N; MN, mineral N; TP, total P; AP, available P. *Q*_T_ is total heat output. The *t*_max_ and *P*_max_ are the time and power to reach the maximum of the peak, respectively. *k* is the growth rate constant. *Q*_T_/*t* is rate of heat output.

## References

[b1] Falkowski P. G., Fenchel T. & Delong E. F. The microbial engines that drive Earth’s biogeochemical cycles. Science 320, 1034–1039 (2008). 18497287 10.1126/science.1153213

[b2] Fierer N. *et al.* Cross-biome metagenomic analyses of soil microbial communities and their functional attributes. Proc. Natl. Acad. Sci. USA 109, 21390–21395 (2012). 23236140 10.1073/pnas.1215210110 PMC3535587

[b3] Talbot J. M. *et al.* Endemism and functional convergence across the North American soil mycobiome. Proc. Natl. Acad. Sci. USA 111, 6341–6346 (2014). 24733885 10.1073/pnas.1402584111 PMC4035912

[b4] Hanson C. A., Fuhrman J. A., Horner-Devine M. C. & Martiny J. B. H. Beyond biogeographic patterns: processes shaping the microbial landscape. *Nat. Rev. Microbiol*. 10, 497–506 (2012). 22580365 10.1038/nrmicro2795

[b5] van der Gast C. J. Microbial biogeography: the end of the ubiquitous dispersal hypothesis? Environ. Microbiol. 17, 544–546 (2015). 25521363 10.1111/1462-2920.12635

[b6] Ge Y. *et al.* Differences in soil bacterial diversity: driven by contemporary disturbances or historical contingencies? ISME J. 2, 254–264 (2008). 18239609 10.1038/ismej.2008.2

[b7] Hu H. W. *et al.* The large-scale distribution of ammonia oxidizers in paddy soils is driven by soil pH, geographic distance, and climatic factors. Front. Microbiol. 6, 938 (2015). 26388866 10.3389/fmicb.2015.00938 PMC4559657

[b8] Fierer N. & Jackson R. B. The diversity and biogeography of soil bacterial communities. Proc. Natl. Acad. Sci. USA 103, 626–631 (2006). 16407148 10.1073/pnas.0507535103 PMC1334650

[b9] Griffiths R. I. *et al.* The bacterial biogeography of British soils. Environ. Microbiol. 13, 1642–1654 (2011). 21507180 10.1111/j.1462-2920.2011.02480.x

[b10] Lauber C. L., Hamady M., Knight R. & Fierer N. Pyrosequencing-based assessment of soil pH as a predictor of soil bacterial community structure at the continental scale. Appl. Environ. Microbio. 75, 5111–5120 (2009). 10.1128/AEM.00335-09 PMC2725504 19502440

[b11] Pellissier L. *et al.* Soil fungal communities of grasslands are environmentally structured at a regional scale in the Alps. Mol. Ecol. 23, 4274–4290 (2014). 25041483 10.1111/mec.12854

[b12] Drenovsky R. E., Steenwerth K. L., Jackson L. E. & Scow K. M. Land use and climatic factors structure regional patterns in soil microbial communities. Global. Ecol. Biogeogr. 19, 27–39 (2010). 10.1111/j.1466-8238.2009.00486.x PMC3891896 24443643

[b13] Green J. L., Bohannan B. J. M. & Whitaker R. J. Microbial biogeography: From taxonomy to traits. Science 320, 1039–1043 (2008). 18497288 10.1126/science.1153475

[b14] Nannipieri P. *et al.* Microbial diversity and soil functions. Eur. J. Soil. Sci. 54, 655–670 (2003).

[b15] Allison S. D. & Martiny J. B. H. Resistance, resilience, and redundancy in microbial communities. Proc. Natl. Acad. Sci. USA 105, 11512–11519 (2008). 18695234 10.1073/pnas.0801925105 PMC2556421

[b16] Rousk J., Brookes P. C. & Bååth E. Contrasting soil pH effects on fungal and bacterial growth suggest functional redundancy in carbon mineralization. Appl. Environ. Microb. 75, 1589–1596 (2009). 10.1128/AEM.02775-08 PMC2655475 19151179

[b17] Hartman W. H. & Richardson C. J. Differential nutrient limitation of soil microbial biomass and metabolic quotients (qCO_2_): is there a biological stoichiometry of soil microbes. PloS One 8, e57127 (2013). 23526933 10.1371/journal.pone.0057127 PMC3602520

[b18] Brockett B. F., Prescott C. E. & Grayston S. J. Soil moisture is the major factor influencing microbial community structure and enzyme activities across seven biogeoclimatic zones in western Canada. *Soil. Biol. Biochem*. 44, 9–20 (2012).

[b19] Leff J. W. *et al.* Consistent responses of soil microbial communities to elevated nutrient inputs in grasslands across the globe. Proc. Natl. Acad. Sci. USA 112, 10967–10972 (2015). 26283343 10.1073/pnas.1508382112 PMC4568213

[b20] Carbonero F., Oakley B. B. & Purdy K. J. Metabolic flexibility as a major predictor of spatial distribution in microbial communities. PloS One 9, e85105 (2014). 24465487 10.1371/journal.pone.0085105 PMC3897421

[b21] Pietri J. A. & Brookes P. Substrate inputs and pH as factors controlling microbial biomass, activity and community structure in an arable soil. *Soil. Biol. Biochem*. 41, 1396–1405 (2009).

[b22] Reth S., Reichstein M. & Falge E. The effect of soil water content, soil temperature, soil pH-value and the root mass on soil CO_2_ efflux–a modified model. Plant Soil 268, 21–33 (2005).

[b23] Martiny J. B. H. *et al.* Microbial biogeography: putting microorganisms on the map. Nat. Rev. Microbiol. 4, 102–112 (2006). 16415926 10.1038/nrmicro1341

[b24] Mäder P. *et al.* Soil fertility and biodiversity in organic farming. Science 296, 1694–1697 (2002). 12040197 10.1126/science.1071148

[b25] Critter S. A., Freitas S. S. & Airoldi C. Microcalorimetric measurements of the metabolic activity by bacteria and fungi in some Brazilian soils amended with different organic matter. Thermochim. Acta 417, 275–281 (2004).

[b26] Hassan W., Chen W., Cai P. & Huang Q. Estimation of enzymatic, microbial, and chemical properties in Brown soil by microcalorimetry. J. Therm. Anal. Calorim. 116, 969–988 (2014).

[b27] Sparling G. Estimation of microbial biomass and activity in soil using microcalorimetry. J. Soil. Sci. 34, 381–390 (1983).

[b28] Burns R. G. *et al.* Soil enzymes in a changing environment: Current knowledge and future directions. Soil. Biol. Biochem. 58, 216–234 (2013).

[b29] Blagodatskaya E. & Kuzyakov Y. Active microorganisms in soil: critical review of estimation criteria and approaches. Soil. Biol. Biochem. 67, 192–211 (2013).

[b30] De Nobili M., Contin M., Mondini C. & Brookes P. C. Soil microbial biomass is triggered into activity by trace amounts of substrate. Soil. Biol. Biochem. 33, 1163–1170 (2001).

[b31] Wadsö I. Characterization of microbial activity in soil by use of isothermal microcalorimetry. *J. Therm. Anal. Calorim*. 95, 843–850 (2009).

[b32] Ahamadou B. *et al.* Microcalorimetric assessment of microbial activity in long-term fertilization experimental soils of Southern China. FEMS Microbiol. Ecol. 70, 186–195 (2009). 10.1111/j.1574-6941.2009.00753.x 19702873

[b33] Barros N., Salgado J. & Feijóo S. Calorimetry and soil. Thermochim. Acta 458, 11–17 (2007).

[b34] Sparling G. Microcalorimetry and other methods to assess biomass and activity in soil. Soil. Biol. Biochem. 13, 93–98 (1981).

[b35] Núñez-Regueira L., Proupín-Castiñeiras J., Rodríguez-Añón J., Villanueva-López M. & Núñez-Fernández O. Design of an experimental procedure to assess soil health state. *J. Therm. Anal. Calorim*. 85, 271–277 (2006).

[b36] Zheng S. *et al.* Soil microbial activity measured by microcalorimetry in response to long-term fertilization regimes and available phosphorous on heat evolution. Soil. Biol. Biochem. 41, 2094–2099 (2009).

[b37] Thomson B. C. *et al.* Soil conditions and land use intensification effects on soil microbial communities across a range of European field sites. Soil. Biol. Biochem. 88, 403–413 (2015).

[b38] Brady N. C. & Weil R. R. (eds) The nature and properties of soils (13th ed.) Ch. 9, 363–411 (Prentice Hall, NJ, 2002).

[b39] Wittmann C., Kähkönen M. A., Ilvesniemi H., Kurola J. & Salkinoja-Salonen M. S. Areal activities and stratification of hydrolytic enzymes involved in the biochemical cycles of carbon, nitrogen, sulphur and phosphorus in podsolized boreal forest soils. Soil. Biol. Biochem. 36, 425–433 (2004).

[b40] DeForest J. L. & Scott L. G. Available organic soil phosphorus has an important influence on microbial community composition. Soil. Sci. Soc. Am. J. 74, 2059–2066 (2010).

[b41] Lauber C. L., Strickland M. S., Bradford M. A. & Fierer N. The influence of soil properties on the structure of bacterial and fungal communities across land-use types. Soil. Biol. Biochem. 40, 2407–2415 (2008).

[b42] Wadsö I. Isothermal microcalorimetry in applied biology. Thermochim. Acta 394, 305–311 (2002).

[b43] Barros N., Gomez-Orellana I., Feijóo S. & Balsa R. The effect of soil moisture on soil microbial activity studied by microcalorimetry. Thermochim. Acta 249, 161–168 (1995).

[b44] Barros N., Feijoó S. & Balsa R. Comparative study of the microbial activity in different soils by the microcalorimetric method. Thermochim. Acta 296, 53–58 (1997).

[b45] Drenovsky R. E., Elliott G. N., Graham K. J. & Scow K. M. Comparison of phospholipid fatty acid (PLFA) and total soil fatty acid methyl esters (TSFAME) for characterizing soil microbial communities. Soil. Biol. Biochem. 36, 1793–1800 (2004).

[b46] Bossio D. & Scow K. Impacts of carbon and flooding on soil microbial communities: phospholipid fatty acid profiles and substrate utilization patterns. Microb. Ecol. 35, 265–278 (1998). 9569284 10.1007/s002489900082

[b47] Wu Y. *et al.* Effects of different soil weights, storage times and extraction methods on soil phospholipid fatty acid analyses. Geoderma 150, 171–178 (2009).

[b48] Frostegård Å. & Bååth E. The use of phospholipid fatty acid analysis to estimate bacterial and fungal biomass in soil. Biol. Fert. Soils 22, 59–65 (1996).

[b49] Rousk J. *et al.* Soil bacterial and fungal communities across a pH gradient in an arable soil. ISME J. 4, 1340–1351 (2010). 20445636 10.1038/ismej.2010.58

[b50] Zogg G. P. *et al.* Compositional and functional shifts in microbial communities due to soil warming. Soil. Sci. Soc. Am. J. 61, 475–481 (1997).

[b51] Frostegård Å., Tunlid A. & Bååth E. Phospholipid fatty acid composition, biomass, and activity of microbial communities from two soil types experimentally exposed to different heavy metals. Appl. Environ. Microb. 59, 3605–3617 (1993). 10.1128/aem.59.11.3605-3617.1993 PMC182506 16349080

