# Supplementary material for: Soil pH, total phosphorus, climate and distance are the major factors influencing microbial activity at a regional spatial scale
Source: Sci Rep. 2016 May 12;6:25815. doi: 10.1038/srep25815 (PMC4864422; doi:10.1038/srep25815)
Supplement: Supplementary Information [file srep25815-s1.pdf]

Soil pH, total phosphorus, climate and distance are the major factors influencing microbial activity at a regional spatial scale

Haichuan Cao[1*], Ruirui Chen[2*], Libing Wang[1*], Lanlan Jiang[1], Fen Yang[3], Shixue Zheng[1#], Gejiao Wang[1] and Xiangui Lin[2]

Table S1 Main soil chemical properties, microbial activities and PLFAs of soil samples from specific location

Sampling sites		Yujiang, Jiangxi	Wuhan, Hubei	Xianning, Hubei	Zhijiang, Hubei	Fengqiu, Henan	Anyang, Henan
**Soil chemical properties**	pH	4.24-5.29	4.35-7.70	7.53-7.85	8.43-8.47	8.51-8.89	8.13-8.19
	SOC (g kg^{-1})	6.32-14.13	13.91-58.28	16.62-18.84	8.68-10.47	5.21-11.47	12.88-14.58
	TN (g kg^{-1})	0.58-1.27	0.73-1.89	1.12-1.85	0.98-1.11	0.51-1.16	1.11-1.20
	SOC/TN	9.37-12.38	6.96-17.89	8.52-15.67	5.14-5.47	8.79-10.25	10.73-13.13
	TP (g kg^{-1})	0.34-0.79	0.29-1.28	0.60-0.79	0.68-0.92	0.79-0.98	1.08-1.26
	SOC/TP	12.78-37.94	17.67-157.51	20.42-30.39	9.43-15.4	5.31-14.49	17.62-23.27
	TN/TP	1.18-2.94	0.11-4.29	1.48-3.03	1.07-1.63	0.52-1.47	0.95-1.03
	MN (mg kg^{-1})	4.08-55.44	48.51-178.45	83.00-106.40	41.74-68.33	2.08-37.37	50.32-85.48
	AP (mg kg^{-1})	2.06-176.35	5.24-218.38	7.85-16.75	2.63-15.79	10.78-39.85	26.01-28.07
	SOC/AP	0.04-5.52	0.15-3.22	1.06-1.92	3.98-0.55	0.13-0.60	0.50-0.52
	TN/AP	0.003-0.53	0.01-0.23	0.10-0.22	0.06-0.42	0.01-0.06	0.04-0.05
**Calorimetric parameters**	P_{max} (μW)	226.03-904.00	200.54-748.91	283.84-548.82	199.85-557.02	472.68-744.57	645.45-654.58
	t_{max} (min)	1032.48-1899.06	2057.5-525.17	725.62-1302.85	888.27-1498.86	515.5-763	778.11-826.48
	Q_T (J g^{-1})	12.93-41.77	7.99-33.77	13.85-33.77	16.87-17.63	9.48-20.17	17.84-17.89
	K (min^{-1})	1.32-7.80	1.34-7.57	3.15-5.18	2.10-5.32	8.1-11.68	5.25-5.43
	Q_T/t (J g^{-1} h^{-1})	0.18-0.53	0.21-0.73	0.33-0.63	0.42-0.43	0.61-1.12	0.44-0.52
**PLFAs** (nmol g^{-1})	Bacteria	0.79-10.99	25.73-31.72	13.38-31.85	21.15	19.62-39.25	29.15-30.51
	Actinomycetes	0.10-1.95	2.15-4.54	4.42-8.30	1.99	1.14-2.82	2.81-3.26
	Fungi	0.02-0.57	1.61-2.55	0.03-1.38	1.36	6.94-28.51	1.51-1.82
	Total PLFA	2.33-22.72	29.91-38.06	20.59-61.31	24.50	55.22-117.56	35.29-33.78

PLFA, phospholipid fatty acid. Chemical properties SOC, soil organic C; TN, total N; MN, mineral N; TP, total P; AP, available P. Q_T is total heat output. The P_{max} and t_{max} are the time and power to reach the maximum of the peak, respectively. k is the growth rate constant. Q_T/t is rate of heat output.

Table S2 Regression analysis that the variability along with NMDS axes explained by environmental factors (soil chemical properties and climate)

		Calorimetric NMDS1 (75%)		Calorimetric NMDS2 (24%)		PLFA NMDS1 (86%)		PLFA NMDS2 (14%)	
		R^2	*P*	R^2	*P*	R^2	*P*	R^2	*P*
**Soil chemical properties**	pH	**0.54**	<0.001	0.64	<0.001	**0.58**	<0.001	0.28	<0.001
	SOC	--	>0.05	0.29	<0.001	**0.42**	<0.001	--	>0.05
	TN	--	>0.05	0.24	<0.001	0.33	<0.001	--	>0.05
	MN	--	>0.05	0.46	<0.001	**0.54**	<0.001	--	>0.05
	TP	**0.48**	<0.001	0.06	<0.05	--	>0.05	0.24	<0.001
	AP	--	>0.05	--	>0.05	--	>0.05	--	>0.05
	SOC/TN	--	<0.001	--	>0.05	0.11	<0.01	0.21	<0.001
	SOC/TP	0.08	=0.02	0.08	<0.05	0.38	<0.001	0.13	<0.01
	SOC/AP	0.22	<0.001	0.10	=0.0045	0.22	<0.001	--	>0.05
	TN/TP	0.09	=0.006	0.1	=0.002	0.25	<0.001	0.19	<0.001
	TN/AP	0.23	<0.001	0.09	=0.009	0.2	<0.001	--	>0.05
**Climate**	MAT	**0.61**	<0.001	0.14	=0.001	**0.63**	<0.001	0.36	<0.001
	MAR	**0.56**	<0.001	0.45	<0.001	**0.54**	<0.001	0.46	<0.001

NMDS, Nonmetric Multidimensional Scaling. PLFA, phospholipid fatty acid. Chemical properties SOC, soil organic C; TN, total N; MN, mineral N; TP, total P; AP, available P. MAT, mean annual temperature; MAR, mean annual rainfall.
